# The association of previously reported polymorphisms for microvascular complications in a meta-analysis of diabetic retinopathy

**DOI:** 10.1007/s00439-014-1517-2

**Published:** 2014-12-07

**Authors:** S. Mohsen Hosseini, Andrew P. Boright, Lei Sun, Angelo J. Canty, Shelley B. Bull, Barbara E. K. Klein, Ronald Klein, The DCCT/EDIC Research Group, Andrew D. Paterson

**Affiliations:** 1 Genetics and Genome Biology Program, The Hospital for Sick Children, Peter Gilgan Centre for Research and Learning, Rm 12.9835, 686 Bay Street, Toronto, ON M5G 0A4 Canada; 2 Institute of Medical Science, University of Toronto, Toronto, ON Canada; 3 LMC Diabetes and Endocrinology, Toronto, ON Canada; 4 Department of Medicine, University of Toronto, Toronto, ON Canada; 5 Dalla Lana School of Public Health, University of Toronto, Toronto, ON Canada; 6 Department of Statistical Sciences, University of Toronto, Toronto, ON Canada; 7 Department of Mathematics and Statistics, McMaster University, Hamilton, ON Canada; 8 Lunenfeld–Tanenbaum Research Institute, Mount Sinai Hospital, Toronto, ON Canada; 9 Department of Ophthalmology and Visual Sciences, University of Wisconsin, Madison, WI USA

## Abstract

We investigated the association of signals from previous GWAS and candidate gene meta-analyses for diabetic retinopathy (DR) or nephropathy (DN), as well as an *EPO* variant in meta-analyses of severe (SDR) and mild diabetic retinopathy (MDR). Meta-analyses of SDR (≥severe non-proliferative diabetic retinopathy (NPDR) or history of panretinal photocoagulation) and MDR (≥mild NPDR), defined based on seven-field stereoscopic fundus photographs, were performed in two well-characterized type 1 diabetes (T1D) cohorts: the Diabetes Control and Complications Trial/Epidemiology of Diabetes Interventions and Complications (DCCT/EDIC, *n* = 1,304) and Wisconsin Epidemiologic Study of Diabetic Retinopathy (WESDR, *n* = 603). Among 34 previous signals for DR, after controlling for multiple testing, no association was replicated in our meta-analyses. rs1571942 and rs12219125 at *PLXDC2* locus showed nominally significant (<0.05) association with SDR in the same direction as previous report, as did rs1801282 in *PPARG* gene with MDR. Among 55 loci previously associated with DN, three showed suggestive associations with SDR in our study without maintaining significance after correction for multiple testing. Of particular interest, rs1617640 (*EPO*) was not significantly associated with DR status, combined SDR–DN phenotype, time to SDR or time to DN (all *P* > 0.05). Lack of replication of previous DR hits and *EPO* despite reasonable statistical power implies that many of these may be false positives. Consistent with pleiotropy, we provide suggestive collective evidence for association between DR and variants previously associated with DN without reaching statistical significance at any single locus.

**Electronic supplementary material:**

The online version of this article (doi:10.1007/s00439-014-1517-2) contains supplementary material, which is available to authorized users.

## Introduction

Microvascular complications of diabetes are major sources of morbidity worldwide. Diabetic retinopathy (DR) is the main cause of blindness in adults of working age (Centers for Disease Control and Prevention 2011) and diabetic nephropathy (DN) is the leading cause of renal failure (Atkins 2005). Extensive pathophysiological and epidemiologic studies over the past two decades have identified risk factors and improved primary or secondary prevention of diabetic complications. Introduction of intensive glycemic control and photocoagulation treatment for DR are examples of the success of this approach. Similarly, the identification of genetic factors for diabetic complications has potential benefit for understanding mechanisms that may lead to better diagnosis, screening or treatment.

Several lines of evidence suggest a genetic contribution to the risk of microvascular complications. In particular, significant familial clustering of both DN and severe DR (SDR) has been reported after accounting for cross-sectional conventional risk factors (The DCCT Research Group 1997). Nonetheless, genome-wide association studies (GWAS), the main approach for investigating genetic basis of complex diseases, have been generally unsuccessful in identifying SNPs showing genome-wide significant (*P* ≤ 5 × 10^−8^) association with DR (Dudbridge and Gusnanto 2008). Besides, top signals from these studies have not been replicated (*P* < 0.05 in the same direction for the same phenotype) (Chanock et al. 2007) in independent populations (Fu et al. 2010; Grassi et al. 2011; Huang et al. 2011; Sheu et al. 2013). GWAS of DN have had moderate success in finding significant signals (*P* ≤ 5 × 10^−8^), yet these studies also suffer from a general lack of robust replication (Brennan et al. 2013). With a few exceptions, candidate gene association studies of diabetic complications have also been similarly unsuccessful in identifying replicable signals. Moreover, undetectable population stratification (and other technical issues) makes candidate gene studies prone to spurious results.

As microvascular complications, DR and DN share pathophysiological mechanisms and common risk factors. However, thus far, few genetic polymorphisms with pleiotropic effects have been reported for these two complications (Abhary et al. 2009; Mooyaart et al. 2011; Tian et al. 2011). Interestingly, some DN studies require the presence of DR in controls resulting in a bivariate outcome.

In the current meta-analysis of DR, we sought to replicate the association of top signals from previous GWAS and meta-analyses of candidate gene studies for DR. Considering possible pleiotropy of DN and DR, we also examined variants associated with DN from previous GWAS and candidate gene meta-analyses for association with DR. Finally, we tested an *EPO* promoter polymorphism (rs1617640), which has been associated with a composite phenotype of end-stage renal disease (ESRD) and proliferative diabetic retinopathy (PDR) (Tong et al. 2008).

## Materials and methods

### Literature review

#### Retinopathy signals

A PubMed search was performed using the following keywords to identify genome-wide association studies for DR: (((diabetes OR diabetic) AND retinopathy)) AND ((genome wide association) OR GWAS). Of 29 identified records (Nov 7, 2013), seven were relevant (Table S1). This included four previous GWAS of DR, a GWAS of retinopathy in individuals without diabetes and two large candidate gene studies with considerable genomic coverage.

Meta-analyses of previous candidate gene association studies were identified by the following PubMed search: ((((genetics OR genetic OR gene OR polymorphism OR SNP OR allele OR genotype OR variant OR variation OR mutation)) AND ((diabetes OR diabetic) AND retinopathy))) AND (meta-analysis). Among 44 records (Mar 24, 2014), 18 were relevant meta-analyses with positive results (Table S3).

#### Nephropathy signals

To identify GWAS studies for DN, we did a PubMed search with the following terms: ((genome wide association OR genome-wide association OR gwas)) AND ((diabetes OR diabetic) AND nephropathy). Out of 163 records (Nov 13, 2013), 11 were relevant (Table S2). We also included hits reported in a previous meta-analysis of candidate gene studies for DN (Mooyaart et al. 2011).

### Study populations

All the studies were conducted in accordance with the declaration of Helsinki, were approved by the institutional ethics review boards and obtained written informed consents from the participants.

#### DCCT/EDIC

The Diabetes Control and Complications Trial (DCCT, 1983–1993) was a randomized clinical trial designed to compare the effect of intensive glycemic management, aimed at normalizing blood glucose, with that of the contemporary conventional diabetes treatment in preventing the development (primary cohort) or slowing the progression (secondary cohort) of DR in type 1 diabetes (T1D) (The DCCT Research Group 1993). Following termination of the DCCT, the majority of participants have been followed in the Epidemiology of Diabetes Interventions and Complications (EDIC) study (White et al. 2010). Subjects underwent frequent seven-field stereoscopic fundus photography during DCCT (every 6 months) and EDIC (an average of 3.8 photos per person over 10 years). Other phenotypic measures, including A1C values, have also been recorded longitudinally during these studies.

#### WESDR

The Wisconsin Epidemiologic Study of Diabetic Retinopathy (WESDR) is a population-based observational cohort study of diabetic patients from southern Wisconsin. We studied T1D patients in WESDR. Patients underwent stereoscopic fundus photography of seven standard fields at baseline and as part of their follow-up examinations performed approximately at 4, 10, 14 and 25 years from baseline. At each follow-up visit, A1C was also measured (Klein et al. 2008).

### Phenotype definition

We defined severe diabetic retinopathy (SDR) as severe non-proliferative diabetic retinopathy (NPDR) or worse. In both studies retinopathy status for each patient was ascertained at the last follow-up visit. Fundus photographs from the DCCT/EDIC and WESDR cohorts were respectively graded in the Fundus Photographic Reading Center and the Ocular Epidemiology Reading Center (both at Department of Ophthalmology in the University of Wisconsin), based on the Early Treatment Diabetic Retinopathy Study (ETDRS) severity scale (Klein et al. 2008; The DCCT Research Group 1995). Severe diabetic retinopathy (SDR) was defined as an ETDRS level of 53/<53 or worse or a history of panretinal photocoagulation (scatter laser) treatment. Participants without retinopathy or with milder forms of retinopathy not reaching this level were considered as controls.

To ensure consistency with some previous studies that may have used a milder endpoint as phenotype, we also used mild diabetic retinopathy (MDR) as an alternate outcome. MDR was defined as the occurrence of mild NPDR or worse. Cases had an ETDRS level above 35/<35 in the DCCT/EDIC and above 31/<31 in WESDR, while controls were below this level (i.e. no retinopathy or only microaneurysms).

In the combined SDR–DN analysis for the *EPO* variant, the case–control status of each participant was determined at the final follow-up visit. Cases were patients with SDR (as above) plus DN: receiving renal replacement therapy (transplant or hemodialysis) or showing gross proteinuria in urinalysis (WESDR) or persistent macroalbuminuria (DCCT/EDIC: AER >300 mg/24 h) or a creatinine-based estimated GFR less than 15 mL/min/1.73 m^2^ (DCCT/EDIC). Controls were patients with no retinopathy or only microaneurysms (ETDRS step <4) and no DN: no history of renal replacement therapy or proteinuria (WESDR) plus being normoalbuminuric (AER <30 mg/24 h) with an e-GFR greater than 60 mL/min/1.73 m^2^ (DCCT/EDIC).

### Genotyping

DCCT/EDIC and WESDR subjects were genotyped using the Illumina (Illumina^®^ Inc., San Diego, CA, USA) Human 1M and HumanOmni1-Quad assays, respectively. Genotypes were called per study using Illumina’s proprietary GenCall algorithm implemented in BEADSTUDIO/GENOMESTUDIO software (Illumina^®^). Genotype calls were exported to PLINK v1.07 (http://pngu.mgh.harvard.edu/purcell/plink/) for further analysis. Extensive QC measures were undertaken to remove samples with low genotyping quality and individuals with cryptic relatedness or ethnic admixture (Paterson et al. 2009). All analyses were limited to individuals from a white European ancestry who clustered with CEU and TSI samples of HapMap 3 (The International HapMap 3 Consortium 2010) in principal component analysis. As another measure against population stratification, the association between the first three principal components (PC) and the outcome was assessed using logistic regression which was not significant in either univariate or multivariate models.

### Imputation

Genotype imputation for untyped SNPs was performed separately for each study using Howie et al. (2009) methodology implemented in impute v2 (http://mathgen.stats.ox.ac.uk/impute/impute_v2.html). Phased autosomal chromosomes from HapMap 3 release 2 and HapMap 2 release 24 were used as reference panels. A multi-population reference panel, consisting of all populations in HapMap, was used for imputation, allowing the software to choose the best customized reference set for each individual. Initial pre-phasing of each study population was performed using shapeit program (http://www.shapeit.fr/) to produce best-guess haplotypes before imputing untyped genotypes into the estimated haplotypes. For the SNPs present in both HapMap releases, HapMap 3 was used as reference. SNPs with low imputation quality (info <0.8) were excluded from further analysis.

### Statistical analysis

#### DR meta-GWAS

We performed GWAS of SDR assuming an additive coding for the genotypes and using dosages from genotype imputation for untyped SNPs in snptest v2.4.1 (https://mathgen.stats.ox.ac.uk/genetics_software/snptest/snptest.html), fitting a logistic regression model adjusting for the effects of age, gender, diabetes duration and mean A1C. Four separate GWAS analyses were performed: one in WESDR and three in cohort–treatment subgroups of DCCT/EDIC (primary cohort, secondary cohort on conventional treatment, secondary cohort receiving intensive treatment). To avoid sparseness, the primary cohort of DCCT/EDIC was not divided into separate treatment groups; but a treatment indicator was added to the logistic model. Meta-analysis was performed at each SNP to combine the results of separate studies using metal (http://www.sph.umich.edu/csg/abecasis/Metal/) to apply inverse-variance weighted methodology assuming fixed effects. Analyses for MDR were performed similarly, but DCCT/EDIC was divided into four subgroups based on cohort and treatment. Here, we present only the results for previous DR and DN signals; complete results of meta-GWAS will be published separately.

#### Association analysis for EPO polymorphism

Association analyses were performed in sas v9.3. For time-to-SDR analysis we tested the association of the variant with time since the onset of study to the first development of SDR using complementary log-log model in each study before combining the results in a fixed-effect meta-analysis. For time-to-DN analysis, we tested the association between *EPO* polymorphism and time to the development of DN using discrete time Cox regression in WESDR and DCCT/EDIC before combining the results in a fixed-effect meta-analysis. All the models adjusted for age and diabetes duration at baseline, gender and a time-dependent updated mean A1C. For ordinal logistic regression, association of *EPO* polymorphism with DR status was tested after removing DN cases, considering a hierarchy of PDR vs. NPDR vs. no-DR.

#### Power calculations and multiple testing

Results with a two-sided *P* < 0.05 after Bonferroni correction for the effective number of tests were considered significant. The effective number of tests was calculated from the combined WESDR and DCCT/EDIC (best-guess) genotypes, using the Genetic Type I error calculator (http://statgenpro.psychiatry.hku.hk/gec/index.php) (Li et al. 2012). Power calculations were conducted using quanto v1.2.4 (http://hydra.usc.edu/gxe/) assuming a log-additive model.

#### Testing collective association of loci with SDR

To evaluate collective association of loci with SDR, we used Fisher’s method for combining independent tests and Stouffer’s *Z* score method (Fisher 1932; Stouffer et al. 1949). In Fisher’s method, *P* values for association at all loci (single SNP at each locus) were combined irrespective of the direction of effect, while in Stouffer’s method, *Z* scores for association were summed considering consistency of direction with the original report.

## Results

Table 1 summarizes the characteristics of participating studies in the current meta-analysis.

**Table 1:** Characteristics of study subjects in DCCT/EDIC and WESDR

Characteristic	Unit	DCCT/EDIC			WESDR
		Primary cohort	Secondary cohort conventional treatment	Secondary cohort intensive treatment	
N^a^					
Total		651	323	330	603
Case SDR		53	114	42	309
Control SDR		598	209	288	294
Gender	% Male	52	54	55	50
Age at final follow-up visit	year	42.0 ± 7.9	43.4 ± 6.9	44.3 ± 7.1	48.5 ± 10.6
Duration of diabetes at final visit	year	18.2 ± 3.2	25.1 ± 4.7	25.6 ± 4.5	34.3 ± 8.5
A1C level at first visit	%	8.81 ± 1.67	8.83 ± 1.50	8.97 ± 1.44	9.93 ± 1.88
	mmol/mol	72.8	73.0	74.5	85.0
Updated mean A1C during study	%	8.23 ± 1.16	8.52 ± 1.06	7.68 ± 0.96	8.91 ± 1.18
	mmol/mol	66.4	69.6	60.4	73.9
Follow-up duration	year	15.6 ± 2.8	16.5 ± 3.0	16.8 ± 2.5	22.0 ± 5.9
Number of fundus photography visits	count	15.6 ± 3.7	17.5 ± 3.4	17.9 ± 3.4	4.5 ± 0.8

Mean ± standard deviation is reported

^a^Number of subjects with phenotype and genotype data after QC

### Replication of previous signals for DR

We investigated the association of previously reported signals for retinopathy with SDR in our meta-analysis. The list for replication comprised three different sources:

1. Top reported signals (*P* < 10^−5^, 54 SNPs in 11 loci) in previous GWAS studies of DR including two large candidate gene association studies with genome-wide coverage (Roy et al. 2009; Sobrin et al. 2011) (Table S1).
2. Top reported signals (*P* < 10^−5^, 22 SNPs in 11 loci) in a previous GWAS of retinopathy in individuals without diabetes (Jensen et al. 2013).
3. Polymorphisms with evidence for association with DR (*P* < 0.05, 18 SNPs in 16 loci) from previous meta-analyses of candidate gene association studies (Table S3).

There was no overlap between the above categories either based on the SNP or the closest gene. The final list included 95 SNPs representing 39 loci. Of these, 87 SNPs representing 32 loci were genotyped or imputed in our data and we identified suitable proxies (*r* ^2^ > 0.9) for 3 more SNPs using genotype data from HapMap and 1,000 genomes. No suitable tag SNPs could be identified for five SNPs (5 loci) including the aldose reductase (CA)_*n*_ repeat polymorphism (Abhary et al. 2009). Among the 90 tested SNPs at 34 independent loci, three SNPs (2 loci) showed nominally significant (*P* < 0.05) association with SDR in our meta-analysis (Table 2). Only SNPs at the *PLXDC2* locus on chromosome ten showed nominal association in the same direction as the original study without maintaining significance after correction for the effective number of tests (*P* > 0.0015 for 34 DR loci). Interestingly, both SNPs at this locus had the same direction of effect in WESDR and DCCT/EDIC subgroups without significant evidence of heterogeneity of effect (*I* ^2^ = 0, *P* > 0.67 Cochran’s *Q* test).

**Table 2:** Variants showing nominal association with DR (*P*_uncorrected_ < 0.05) in the current study among the SNPs previously associated with DR

Phenotype	SNP	Chr	Position	A1	A2	Original association			Closest gene	Meta-analysis (WESDR + DCCT/EDIC)					Discovery vs. replication
						OR	*P*	References		FRQ	OR (95 % CI)	*P*_meta_*	Direction	*P*_Het_	
SDR	rs39059	7	29,221,995	A	G	1.29	2.6E−04	Hu et al. (2011)	*CHN2*	0.63	0.78 (0.65–0.94)	8.6E−03	−−−−	0.36	+−
SDR	rs1571942	10	20,582,640	A	G	0.60	3.5E−07	Huang et al. (2011)	*PLXDC2*	0.85	0.71 (0.55–0.91)	6.4E−03	−−−−	0.67	−−
SDR	rs12219125	10	20,633,093	T	G	1.62	9.3E−09	Huang et al. (2011)	*PLXDC2*	0.15	1.40 (1.10–1.79)	7.2E−03	++++	0.84	++
MDR	rs1801282	3	12,368,125	G	C	0.81	3.0E−02	Ma et al. (2012)	*PPARG*	0.13	0.73 (0.57–0.93)	9.7E−03	−+−−−	0.004	−−

*Chr* chromosome, *Position* physical position based on hg18, *A1/A2* effect/other alleles, *OR* odds ratio for the effect allele, *P* the association *P* value in the original GWAS, *FRQ* effect allele frequency, *P*_*meta*_ *P* value in the case–control meta-analysis unadjusted for multiple testing, *Direction* direction of effect in participating studies in this meta-analysis with this order, *SDR* WESDR, DCCT/EDIC primary cohort, secondary cohort—conventional treatment, secondary cohort—intensive treatment, *MDR* WESDR, DCCT/EDIC PRI cohort—conv. Rx, PRI cohort—intensive Rx, SEC cohort—conv. Rx, SEC cohort—intensive Rx, *P*_*Het*_ *P* value of Cochran’s *Q* test for heterogeneity, *Discovery vs. replication* direction of effect in the original report (discovery) is compared with the current study (replication)

* The significance threshold adjusting for the effective number of test is *P* < 1.5 × 10^−3^

To estimate statistical power to detect association at these loci in the current study, we performed calculations using effect size estimates from the original reports and allele frequencies from our data. Our study was expected to have strong power to detect associations at 25 loci (1 − *β* > 0.85, two-sided *α* = 0.05), moderate power at 6 loci (0.6 < 1 − *β* < 0.8, two-sided *α* = 0.05) and low power at 3 loci (1 − *β* < 0.5). To avoid inflated power estimates due to the winner’s curse, we repeated power calculations after shrinking the original effect sizes by 50 % (Ioannidis et al. 2009; Sun et al. 2011). Based on these conservative bias-reduced calculations, the current study has high power (1 − *β* > 0.8, *α* = 0.05) to detect association at 9 loci, moderate power (0.5 < 1 − *β* < 0.8, *α* = 0.05) for 7 loci, and low power (1 − *β* < 0.5, *α* = 0.05) for the remaining 18 loci.

Considering possible statistical power limitations, we asked whether there is evidence for collective association of these loci with SDR in our meta-analysis. Fisher’s combined probability test did not show statistically significant evidence for association (*P* = 0.54); yet, Stouffer’s *Z* score method which considers the direction of association showed a marginally significant collective evidence for association with SDR at these 34 loci (*P* = 0.04). However, after dropping the top nominally significant locus on chromosome 10, the joint analysis of the remaining loci was not significant (*P* = 0.09). Of the 34 tested loci, the direction of association was consistent between our meta-analysis and the original report at 19 loci not indicating an enrichment (exact binomial test *P* = 0.6). Besides, there was no statistically significant correlation between effect size estimates in our meta-analysis and the original studies for these 34 loci (*r* = 0.12, *P* = 0.38, Pearson’s correlation). Figure S1 compares effect size estimates in our meta-analysis with the original reports.

To ensure consistency with some of the previous studies that may have used milder levels of DR as the outcome, we also investigated the association of previously reported signals for retinopathy with mild diabetic retinopathy (MDR) in our meta-analysis. Among the 90 tested SNPs at 34 independent loci, only a single SNP in *PPARG* showed nominally significant association with MDR (*P* = 0.0096). The direction of effect was consistent with the previous report (Table 2); however, there was significant evidence for heterogeneity of effect in our meta-analysis (*I* ^2^ = 74, *P* = 0.004 Cochran’s *Q* test). The association at this SNP was not significant after adjusting for multiple testing. Similar to SDR analysis, 19 out of 34 loci showed association in a direction consistent with the original reports which is not indicative of an enrichment (exact binomial test *P* = 0.6). After removing the top signal at *PPARG*, there was no significant collective evidence for association with MDR at the remaining 33 loci (*P* = 0.54, Stouffer’s *Z* score method).

### Association of previous DN variants with DR

A list was composed of SNPs with evidence for association with DN based on the following criteria:

1. Top signals from previous GWAS of DN (Table S2, *P* ≤ 10^−4^, 79 SNPs, 49 loci).
2. SNPs with evidence for association with DN in a previous comprehensive meta-analysis of genetic association studies (Mooyaart et al. 2011) (*P* < 0.05, 24 SNPs/loci).

We also included other reported SNPs in proximity of top hits that did not necessarily meet our *P* value threshold in each study. This increased the total number of investigated SNPs to 122 representing 59 loci. Genotype or imputation data were available for 107 SNPs and appropriate proxies (*r* ^2^ > 0.8) were found for eight others based on HapMap 2 and 3 data. Among 115 tested SNPs representing 55 loci, 9 SNPs at 5 independent loci showed nominal evidence (*P* < 0.05) for association with SDR in our meta-analysis (Table 3). This is not significantly higher than the number of successes expected under the null (*P* = 0.14, exact binomial test). After adjusting for the effective number of tests (significance threshold *P* < 6.97 × 10^−4^ for 72 effective tests), no SNP maintained significance for association with SDR; yet SNPs at three loci (near *CPVL/CHN2, PVT1, LINC00523*) showed suggestive association (*P* < 1.39 × 10^−2^ for 72 effective tests). In a similar analysis, none of the 115 tested SNPs showed significant association with MDR (all *P* > 0.05).

**Table 3:** Variants showing nominal association (*P*_uncorrected_ < 0.05) in SDR meta-analysis among SNPs previously associated with diabetic nephropathy

SNP	Chr	Position	Reference	Closest gene	A1/A2	Original association (DN)		Current meta-analysis of SDR				Direction (DN vs DR)
						OR	*P*	Freq1	OR	Direction	*P*_case–control_^*^	
rs11723864	4	174,810,981	Sandholm et al. (2012)	HAND2	C/G	0.66	6.9E−07	0.92	0.66	−++−	0.025	−−
rs39059	7	29,221,995	Pezzolesi et al. (2009)	CPVL/CHN2	A/G	1.39	5.0E−06	0.63	0.78	−−−−	0.009	+−
rs39075	7	29,243,217		CPVL/CHN2	A/G	0.70	6.5E−07	0.40	1.25	+−++	0.016	−+
rs10808565	8	129,076,594	Hanson et al. (2007)	PVT1	T/C	3.34	1.4E−03	0.34	0.77	−−−−	0.007	+−
rs3815871	8	129,077,760		PVT1	C/G	0.36	1.1E−03	0.33	0.77	−−−−	0.009	−
rs1411766	13	109,050,161	Pezzolesi et al. (2009)	IRS2	A/G	1.41	1.8E−06	0.37	1.21	+−++	0.044	++
rs17412858	13	109,050,609		IRS2	A/G	0.71	1.8E−06	0.63	0.82	−+−−	0.044	−−
rs1018534	14	100,189,987	Sandholm et al. (2012)	LINC00523	T/G	0.84	9.1E−05	0.32	0.75	−−−−	0.004	−−
rs1467537	14	100,191,027		LINC00523	T/C	0.82	2.4E−04	0.30	0.75	−−−−	0.006	−−

Association direction in the original study (diabetic nephropathy) and current study (diabetic retinopathy) is compared

*Chr* chromosome, *Position* physical position based on hg18, *A1/A2* effect/other alleles, *OR* odds ratio, *P* original association *P* value with diabetic nephropathy, *Freq1* mean frequency of A1 in the current meta-analysis, *Direction* direction of effect in each study in the current meta-analysis (order: WESDR, primary cohort, secondary cohort—conventional Rx, secondary cohort—intensive Rx of DCCT/EDIC), *P*_case–control_ *P* value in case–control meta-analysis of SDR without adjusting for multiple testing

* The significance threshold after accounting for effective number of tests is *P* < 6.97 × 10^−4^ with suggestive association at *P* < 1.39 × 10^−2^

The current study may be underpowered to detect association of single SNPs with modest effect sizes; therefore, we tested whether the examined DN loci collectively provide evidence for association with SDR. Collectively, these 55 loci showed statistically significant association with SDR (*P* = 0.049, Fisher’s combined probability test). 37 loci showed association with SDR in the same direction as previously reported for DN (*P* = 0.007, exact binomial test). There was also a statistically significant positive correlation between the SDR effect sizes in our meta-analysis and the DN effect sizes in the original reports for these loci (*r* = 0.41, *P* = 0.001, Pearson’s correlation, Figure S2).

### Association of EPO promoter polymorphism with SDR and DN

Table 4 summarizes the meta-analysis results for the *EPO* promoter polymorphism, rs1617640, which did not show significant association with SDR status (*P* = 0.79) or time to SDR (*P* = 0.61). Since rs1617640 was originally associated with a combined ESRD and PDR phenotype (Tong et al. 2008), we also tested its association with a combined DN and SDR phenotype. The SNP was not associated with SDR + DN status at the last follow-up visit in either WESDR or DCCT/EDIC, nor in the meta-analysis of the two populations (*P* = 0.10, *β* = 0.37, 95 % CI: [−0.07, 0.81] in our meta-analysis vs. *β* = −0.41, 95 % CI: [−0.52,−0.29] in the original study). To increase consistency with the original study which required a minimum of 15 years of diabetes in controls, we repeated our analysis with a similar duration criteria which did not change the results (*P* = 0.07, *β* = 0.41, 95 % CI: [−0.04, 0.85] in opposite direction to the original study). Furthermore, rs1617640 was not associated with time to development of nephropathy in either WESDR (*P* = 0.74) or DCCT/EDIC (*P* = 0.065).

**Table 4:** Association of *EPO* promoter polymorphism (rs1617640) with SDR and DN in WESDR and DCCT/EDIC

Analysis	Study	Cases (events)	Controls (censored)	MAF (cases)	MAF (controls)	Beta	S**E**	*P*	*P*_het_	*I* ^2^	Direction
Combined PDR + ESRD	Tong et al. meta-analysis	1,618	954	0.38	0.47	−0.405	0.060	1.9E−11			−−−
Combined SDR + DN	WESDR	163	88	0.40	0.34	0.295	0.277	0.29			
	DCCT/EDIC	41	452	0.43	0.43	0.507	0.381	0.18			
	WESDR + DCCT/EDIC	204	540	0.41	0.42	0.368	0.224	0.10	0.65	0	++
Combined SDR + DN (duration ≥15 years)	WESDR	163	76	0.40	0.35	0.288	0.276	0.30			
	DCCT/EDIC	40	411	0.44	0.44	0.662	0.404	0.10			
	WESDR + DCCT/EDIC	203	487	0.41	0.43	0.406	0.228	0.07	0.44	0	++
SDR status	WESDR + DCCT/EDIC	518	1,389	0.38	0.40	−0.025	0.094	0.80	0.28	21	++−−
Time-to SDR	WESDR + DCCT/EDIC	560	1,347			−0.032	0.064	0.61	0.21	34	−+−−
Time-to DN	WESDR + DCCT/EDIC	279	1,493			0.135	0.093	0.15	0.24	29	++
DR (recessive model)*	WESDR + DCCT/EDIC	PDR	300			0.244	0.140	0.08	0.67	0	−+++
		NPDR	728								
		no-DR	587								

The additive effect (beta) of allele C (minor allele) and its standard error (SE) and two-tail association *P* values (*P*) are presented in the table

For case–control analyses the frequency of minor allele (MAF) in cases and controls is provided

For each meta-analysis Cochran’s heterogeneity *P* value (*P*_het_) and *I* ^2^ are provided

Direction of effect in participating studies are presented with the following order: WESDR, DCCT/EDIC (for combined SDR + DN and time to DN analyses), WESDR, primary cohort, secondary cohort—conventional Rx, secondary cohort intensive Rx of DCCT/EDIC (for SDR analyses), Utah T2D, GoKind and Boston T1D studies (Tong et al. meta-analysis)

Results from the original report of *EPO* polymorphism association with ESRD + PDR (Tong et al. 2008) are provided for comparison

All models in the current study adjust for age, gender, diabetes duration and glycemic exposure measured by A1C

* *DR* ordinal logistic regression with (PDR vs NPDR vs no-DR) as outcome. The model compares CC with AC + AA genotypes. DN cases have been excluded from this analysis

A previous study suggested that *EPO* polymorphism may be associated with DR only under a recessive model (Abhary et al. 2010). We, therefore, tested the association of rs1617640 with DR status (PDR vs NPDR vs no-DR) in a recessive model after removing DN cases; it was not significant (*P* = 0.08 in opposite direction to the original report).

We evaluated the statistical power of the current study to detect association at the *EPO* variant assuming an odds ratio of 0.66 (95 % CI: 0.58–0.76) for the additive effect of protective allele (effect size of the two replication cohorts, GoKinD and Boston T1D, in the original report) and an allele frequency of 0.42. Based on the upper and lower confidence limits of the effect estimate, a study of current size has statistical power between 0.62 and 0.99 to detect an association (two-tailed α level of 0.05).

## Discussion

Despite evidence for a genetic contribution to diabetic retinopathy, GWAS have not been very successful in identifying loci for DR. Neither have the top loci from these studies been replicated in independent populations (Fu et al. 2010; Grassi et al. 2011; Huang et al. 2011; Sheu et al. 2013). Results from candidate gene association studies have also been often conflicting (Abhary et al. 2009).

Previous studies vary considerably in outcome definition and grading methods. In the current study we conducted a meta-analysis of two relatively large T1D cohorts using seven-field stereoscopic fundus photographs, the gold standard method to phenotype DR. We used SDR and MDR as our outcomes. These severity grades represent very severe (preproliferative) and very mild NPDR respectively and together correspond well with the outcomes used in most previous studies. Nevertheless, current evidence supports a genetic contribution mostly for the more severe form of DR.

Among the 34 tested loci, a single locus (2 SNPs) on chromosome 10 showed significant association with SDR in the same direction. The original association between this locus and DR was reported in a case–control GWAS of Taiwanese type 2 diabetes (T2D) patients (*P* = 9.3 × 10^−9^ for rs12219125) as one of the two loci meeting genome-wide significance (Huang et al. 2011). Huang et al. failed to report qq-plot, histogram of *P* value and genomic control inflation factor in their manuscript and their study may suffer from inflation of type I error as six separate genetic models were fitted for each SNP without adjustment for multiple testing. Yet, the direction of the effect at this locus remained consistent with the original study in WESDR and all the DCCT/EDIC subgroups. The two SNPs are located 50 kb apart and are in strong LD (*r* ^2^ = 0.91 in HapMap 3 CEU): rs1571942 is located in the last intron of *PLXDC2*, Plexin domain-containing protein 2, while rs12219125 is located 24 kb downstream of this gene. *PLXDC2*, also referred to as tumor endothelial marker seven-related protein (TEM7R), is implicated in neurogenesis and angiogenesis (Miller-Delaney et al. 2011). Interestingly, a closely related gene, tumor endothelial marker 7 (TEM7), shows strong overexpression in fibrovascular membranes from PDR, suggesting a role in proliferation and maintenance of neovascular endothelial cells (Yamaji et al. 2008).

Aside from *PLXDC2*, none of the other loci suggestive for DR in previous GWAS and candidate gene meta-analyses were replicated in our study. The current study is expected to have reasonable statistical power (>0.5) to detect associations for about half of these loci (16 out of 34), implying that the general lack of replication is unlikely to be a mere sample size issue. After removing the *PLXDC2* locus, the remaining loci do not show collective evidence for association with SDR and there is no correlation between effect sizes in our meta-analysis and the original reports suggesting that many of these loci may not be associated with SDR.

Several factors may have contributed to the general lack of success in identifying replicable signals in association studies for DR. These studies usually do not account for the known DR risk factors (such as diabetes duration or glycemic control) which may have led to reduction in power or potential confounding. Using less accurate methods for defining cases and controls (such as self-report or review of medical records) and differences in phenotype definitions may be other contributing factors. Some of the previous studies were conducted in other ethnic groups which may explain the lack of replication for some loci. Similarly, many of the previous studies examined patients with T2D which may be another factor contributing to the lack of replication, although current pathophysiologic knowledge does not support innate differences in DR between T1D and T2D (Fowler 2008). Besides, replication populations had limited statistical power to detect modest effects considering the winner’s curse. Finally, it is possible that most of the genetic contribution to DR is determined by rare variations not covered in conventional GWAS and candidate gene studies.

Common pathophysiology and a few genetic reports support possible pleiotropy between DN and DR (Abhary et al. 2009; Mooyaart et al. 2011; Tian et al. 2011). In the current study we tested previous suggestive signals for DN for association with SDR. Although none of the loci showed statistically significant association after accounting for multiple testing, three loci showed suggestive association with SDR. Furthermore, there was evidence for collective association of the studied loci with SDR and a positive correlation between the observed effect sizes for SDR and previously reported effect sizes for DN. These observations suggest that this set of DN loci contains association signals for SDR and limited statistical power may be contributing to lack of statistically significant association between single loci and SDR. Yet, most previous DN signals are probably not associated with DR. Besides, the validity of association (with DN) at many of these loci is unconfirmed. A meta-analysis of independent T1D cohorts did not replicate association with DN at most of these loci (Williams et al. 2012).

Despite reasonable statistical power, our study did not provide evidence for association of an *EPO* promoter polymorphism with SDR, DN, or combined SDR–DN phenotype and the directions of effect were opposite to the original report. Time to event analysis is expected to have better statistical power and more clinical relevance in longitudinal studies; however, rs1617640 was also not associated with either time to SDR or time to DN in our meta-analyses.

Elevated vitreal levels of *EPO* in both patients with PDR and animal models have suggested *EPO* as an ischemia-induced angiogenic factor in PDR (Watanabe et al. 2005). Consistent with a role in DR, *EPO* shows overexpression in the retina of diabetic patients without clinically detectable retinopathy compared to non-diabetic controls (Garcia-Ramirez et al. 2008). These observations were followed by a candidate gene association study which examined 10 SNPs in the *EPO* region and identified a significant association between rs1617640 and a combined ESRD + PDR phenotype (Tong et al. 2008). Functional studies suggest a role for rs1617640 in the expression of *EPO* (Tong et al. 2008); however, subsequent studies have failed to provide consistent evidence supporting the association between this SNP and DR. Abhary et al. (2010) observed an association between the CC genotype at this SNP and DR status only in T2D patients (*n* = 345) assuming a recessive model but no association in T1D patients or using an allelic association test. A subsequent candidate gene association study in T2D patients (345 DR and 356 no-DR) did not find an association between rs1617640 and DR in either allelic or recessive models (Balasubbu et al. 2010). Similarly, a meta-analysis of FinnDiane and UK-ROI cohorts did not provide evidence for association of rs1617640 with PDR or with the combined ESRD–PDR phenotype (Williams et al. 2012). It is worth mentioning that unlike the present study, all the populations in the original report (Tong et al. 2008) used an extreme control phenotype (controls free from both DR and ESRD after 10–15 years of diabetes duration). Without any consideration of population stratification in the original report, it is not possible to exclude the possibility of a spurious association. Moreover, ESRD is a strong predictor of PDR (and vice versa) and current evidence for association of rs1617640 and DR independent of ESRD is limited. Overall, our study further calls into question the validity of association between rs1617640 and DR in T1D.

In conclusion, with the possible exception of *PLXDC2*, none of the top hits from previous GWAS and candidate gene meta-analysis for DR were replicated in our meta-analysis. Our study provides suggestive evidence for association of three known DN loci with SDR as further evidence for pleiotropy of microvascular complications. We also did not find evidence supporting the previously reported association between DR and the *EPO* promoter polymorphism (rs1617640). Replication studies of this kind, using well-phenotyped populations with reasonable statistical power, are of increasing importance in establishing or rejecting reported signals from GWAS and candidate gene studies without replication.

## Electronic supplementary material

Below is the link to the electronic supplementary material.

Supplementary material 1 (PDF 783 kb)
