# Supplementary material for: The association of previously reported polymorphisms for microvascular complications in a meta-analysis of diabetic retinopathy
Source: Hum Genet. 2014 Dec 7;134(2):247–57. doi: 10.1007/s00439-014-1517-2 (PMC4291513; doi:10.1007/s00439-014-1517-2)
Supplement: Supplementary file 1 — Supplementary material 1 (PDF 783 kb) [file 439_2014_1517_MOESM1_ESM.pdf]

ONLINE-ONLY SUPPLEMENTAL MATERIAL

Table S1. Summary of genome-wide association studies of diabetic retinopathy

Diabetes type	Discovery N (cases/controls)	Phenotyping	Comparison (case vs control)	Ethnicity	Genotyping platform	Imput -ation	Smallest P-value	Reference
**T2D**	286 (103/183)	fundus photograph 7 field	MS-NPDR and PDR (ETDRS: 43-85) vs normal to early NPDR (ETDRS: 10-37)	Mexican American	Affymetrix 100K	HM3	1.8E-05	(1)
**T2D**	749 (174/575)	fundus exam by ophthalmologist	NPDR or PDR vs no-DR	Taiwanese	Illumina HumanHap550	no	3.0E-15	(2)
**T1D**	all: 973/1856<br>No DN subjects*: 281/1715	GoKinD: self-reported laser Rx<br>EDIC: fundus photograph 7 field	PDR or DME vs not	Caucasian	GoKinD: Affymetrix 5.0<br>EDIC: Illumina 550K	HM2	1.6E-07	(3)
**T2D**	1007 (437/570)	fundus exam by ophthalmologist	PDR vs no DR	Taiwanese	Illumina OmniExpress	HM2	1.3E-07	(4)
**T2D**	1254 (222/1032)	fundus photograph (1-2 field)	any DR (ETDRS ≥14) vs no DR (ETDRS <14)	Caucasian	Illumina iSelect IBC	no	1.1E-06	(5) **
	1154 (122/1032)		DR (ETDRS ≥30) vs no DR (ETDRS <14)				5.3E-07	
**T1D**	437 (128/309)	fundus photograph 7 field	Severe DR vs no severe DR	African American	Illumina GoldenGate 1536 SNPs	no	1.1E-04	(6) **
**None**	19,411 (1122/18,289)	Fundus photograph	Mild retinopathy vs no retinopathy in none diabetics	mixed	Variable	HM2	2.49E-06	(7)

NPDR: nonproliferative DR; MS-NPDR: moderate-to-severe NPDR; PDR: proliferative DR; DME: diabetic macular edema; HM:HapMap

* Secondary analysis after removing subjects with diabetic nephropathy. In this study only the hits from GoKinD analysis were considered for replication.

** These two studies are candidate gene association studies with considerable genomic coverage and not GWAS.

Table S2. Summary of genome-wide association studies of diabetic nephropathy

Reference	Design	Diabetes Type	Discovery N (cases/controls)	Case	Control	Ethnicity / Study	Genotyping	minimum P	Number of loci
(8)	pooled case-control	T1D	1096 (547/549)	ESRD and T1D duration ≥10 yrs	no DN duration >20 yrs	White, GoKinD	Illumina HumanHap 550	1.60E-05	2
(9)	case-control	T1D	1178 (601/577)	DN: persistent proteinuria for >10 yrs or ESRD	no DN duration >20 yrs	White, GoKinD	Affymetrix 10 K Xba	2.12E-05	1
(10)	pooled case-control	T2D	207 (105/102)	ESRD	No macroalbuminuria & diabetes duration ≥10yrs	Gila River Indian	Affymetrix 100k	2.00E-06	1
(11)	case-control	T2D	188 (94/94)	DR and overt DN	DR but no DN	Japanese	100K multiplex PCR-invader assays	8.00E-06	1
(12)	case-control	T2D	188 (94/94)	DR and overt DN	DR but no DN	Japanese	100K multiplex PCR-invader assays	1.40E-06	1
(13)	case-control	T2D	1994 (965/1029)	ESRD and diabetes duration >5 yrs	Healthy controls	African American	Affymetrix array 6.0	7.04E-07	18
(14)	case-control	T2D	1467 (718/749)	DN: persistent proteinuria or ESRD	no DN duration >10 yrs	White, UK GokinD	Illumina Human NS12	2.00E-05	1
(15)	case-control	T1D	1705 (820/885)	DN: ESRD or Proteinuria	no DN duration >15 yrs	White, GoKinD	Affymetrix 500K	5.00E-07	4
(16)	case-control	T1D	6652 (1399/5253)	ESRD	non-ESRD	White	Various, imputation to HM2	2.04E-09	7
	case-control	T1D	6231 (2916/3315)	DN	no DN			2.14E-07	1
	case-control	T1D	4714 (1399/3315)	ESRD	normalbuminuria			3.27E-07	12
(17)	case-control	T2D	188 (94/94)	DR and overt DN	DR but no DN	Japanese	55K multiplex PCR-invader assays	2.00E-05	1
(18)	candidate gene	mixed	613 (374/239)	ESRD and PDR	No ESRD or PDR	White	single SNP genotyping	2.76E-11	1

ESRD: End Stage Renal Disease; DN: diabetic nephropathy; DR: diabetic retinopathy; Number of loci with P<1E-4 is reported

Table S3. List of genes / variants with evidence for association (P < 0.05) with diabetic retinopathy in previous meta-analyses of candidate gene association studies.

gene symbol	variant	rsID	diabetes type	comparison	reference
ACE	I/D	rs4646994	T2D	DR vs DWR	(19)
ACE	I/D	rs4646994	T2D	PDR vs DWR	(19)
ACE	I/D	rs4646994	mixed	DR vs DWR	(20)
ACE	INS/DEL	rs4646994	mixed	PDR vs NPDR	(21)
ACE	I/D	rs4646994	mixed	DR vs DWR	(22)
ACE	I/D	rs4646994	mixed	PDR vs DWR	(22)
AGER	G1704T	rs184003	T2D	DR vs DWR	(23)
AGER	-374T/A	rs1800624	T2D	DR vs DWR	(24)
AGER	Gly82Ser	rs2070600	T2D	DR vs DWR	(24)
AGT	rs4762	rs4762	mixed	DR vs DWR	(21)
AKR1B1	rs759853	rs759853	T1D	DR vs DWR	(21)
AKR1B1	rs759853	rs759853	mixed	PDR vs NPDR	(21)
AKR1B1	(CA)n repeat		mixed	DR vs DWR	(21)
AKR1B1	(CA)n repeat		mixed	NPDR vs DWR	(21)
AKR1B1	(CA)n repeat		mixed	PDR vs DWR	(21)
AKR1B1	(CA)n repeat		mixed	PDR vs NPDR	(21)
AKR1B1	(CA)n repeat		T1D	DR vs DWR	(21)
CHN2	rs39059 +	rs39059	T2D	DR vs DWR	(25)
ICAM1	rs13306430	rs13306430	T2D	DR vs DWR	(21)
ITGA2	rs2910964	rs2910964	T2D	DR vs DWR	(21)
MTHFR	C677T	rs1801133	T2D	DR vs DWR	(26)
MTHFR	C677T	rs1801133	T2D	DR vs DWR	(26)
MTHFR	677C/T	rs1801133	mixed	DR vs DWR	(27)
NOS3	4b/a (27bp indel)	rs3138808	T2D	DR vs DWR	(28)
PPARG	Pro12Ala	rs1801282	T2D	DR vs DWR	(29)
SERPINE1	4G/5G	rs1799768	T2D	DR vs DWR	(30)
SOD2	C47T	rs4880	mixed	DR vs DWR	(31)
SUV39H2	rs17353856	rs17353856	T1D	PDR vs DWR	(32)
VEGFA	-634G>C	rs2010963	T2D	DR vs DWR	(33)
VEGFA	rs2010963	rs2010963	T2D	NPDR vs DWR	(21)
VEGFA	-634 C/G	rs2010963	mixed	NPDR vs DWR	(34)
VEGFA	-460T/C	rs833061	T2D	DR vs DWR	(35)
VEGFA	rs699947	rs699947	mixed	DR vs DWR	(36)

T1D: type 1 diabetes, T2D: type 2 diabetes, DR: diabetic retinopathy, DWR: diabetes without retinopathy, PDR: proliferative diabetic retinopathy, NPDR: non-proliferative diabetic retinopathy

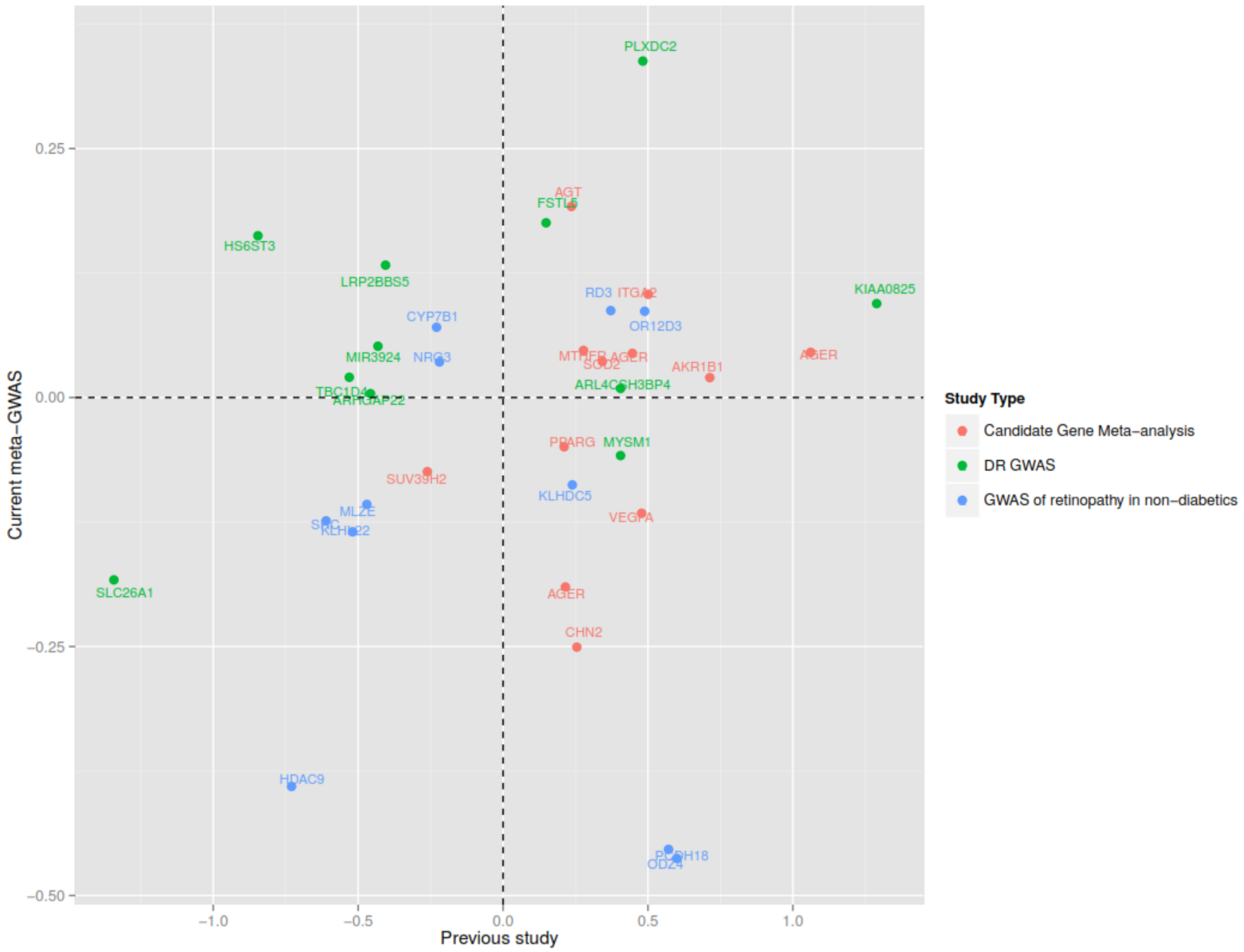

Figure S1. Scatter plot of allelic effect size estimates for DR. For each SNP, effect size in the current meta-GWAS is compared with the previously reported estimate in the discovery study. Only a single SNP from each locus is included and dots are labeled by the closest gene name. Loci are assigned into groups based on the original study type. There is no significant correlation between effect sizes in the current study and what reported in the original study (*r* = 0.12, P = 0.38, Pearson's correlation).

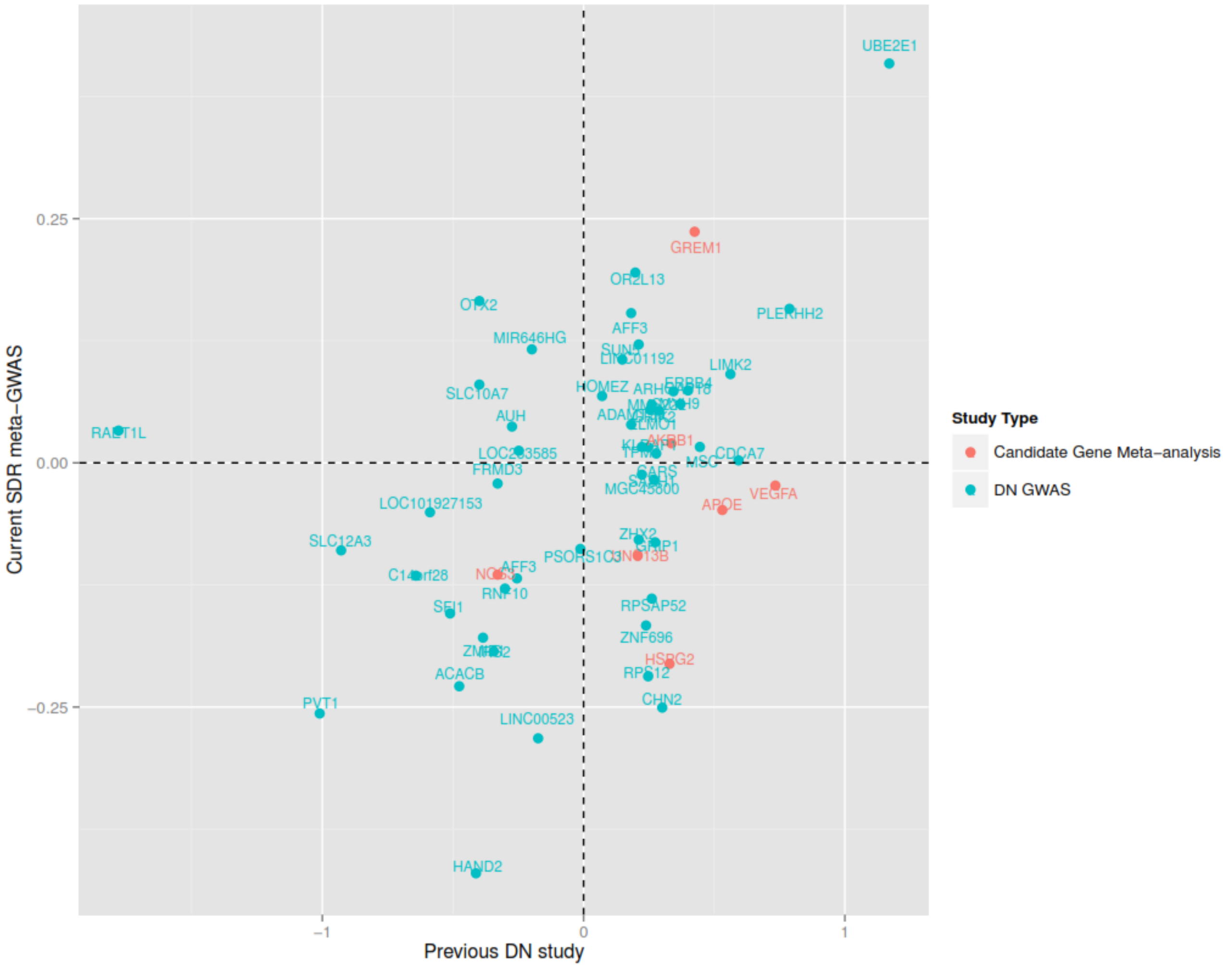

Figure S2. Scatter plot of allelic effect size estimates for DR vs DN. For each SNP, effect size in the current meta-GWAS of SDR is compared with the previously reported estimate in the discovery DN study. Only a single SNP from each locus is included and dots are labeled by the closest gene name. Loci are assigned into groups based on the original study type. There is a statistically significant correlation between effect sizes in the current study and that reported in the original DN genetic association study (r = 0.41, P = 0.001, Pearson's correlation)

References:

1. Fu YP, Hallman DM, Gonzalez VH, Klein BE, Klein R, Hayes MG, Cox NJ, Bell GI, Hanis CL: Identification of Diabetic Retinopathy Genes through a Genome-Wide Association Study among Mexican-Americans from Starr County, Texas. Journal of ophthalmology 2010;2010

2. Huang YC, Lin JM, Lin HJ, Chen CC, Chen SY, Tsai CH, Tsai FJ: Genome-wide association study of diabetic retinopathy in a Taiwanese population. Ophthalmology 2011;118:642-648

3. Grassi MA, Tikhomirov A, Ramalingam S, Below JE, Cox NJ, Nicolae DL: Genome-wide meta-analysis for severe diabetic retinopathy. Hum Mol Genet 2011;20:2472-2481

4. Sheu WH, Kuo JZ, Lee IT, Hung YJ, Lee WJ, Tsai HY, Wang JS, Goodarzi MO, Klein R, Klein BE, Ipp E, Lin SY, Guo X, Hsieh CH, Taylor KD, Fu CP, Rotter JI, Chen YD: Genome-wide association study in a Chinese population with diabetic retinopathy. Human molecular genetics 2013;

5. Sobrin L, Green T, Sim X, Jensen RA, Tai ES, Tay WT, Wang JJ, Mitchell P, Sandholm N, Liu Y, Hietala K, Iyengar SK, Brooks M, Buraczynska M, Van Zuydam N, Smith AV, Gudnason V, Doney AS, Morris AD, Leese GP, Palmer CN, Swaroop A, Taylor HA, Jr., Wilson JG, Penman A, Chen CJ, Groop PH, Saw SM, Aung T, Klein BE, Rotter JI, Siscovick DS, Cotch MF, Klein R, Daly MJ, Wong TY: Candidate gene association study for diabetic retinopathy in persons with type 2 diabetes: the Candidate gene Association Resource (CARe). Invest Ophthalmol Vis Sci 2011;52:7593-7602

6. Roy MS, Hallman DM, Fu YP, Machado M, Hanis CL: Assessment of 193 candidate genes for retinopathy in African Americans with type 1 diabetes. Archives of ophthalmology 2009;127:605-612

7. Jensen RA, Sim X, Li X, Cotch MF, Ikram MK, Holliday EG, Eiriksdottir G, Harris TB, Jonasson F, Klein BE, Launer LJ, Smith AV, Boerwinkle E, Cheung N, Hewitt AW, Liew G, Mitchell P, Wang JJ, Attia J, Scott R, Glazer NL, Lumley T, McKnight B, Psaty BM, Taylor K, Hofman A, de Jong PT, Rivadeneira F, Uitterlinden AG, Tay WT, Teo YY, Seielstad M, Liu J, Cheng CY, Saw SM, Aung T, Ganesh SK, O'Donnell CJ, Nalls MA, Wiggins KL, Kuo JZ, van Duijn CM, Gudnason V, Klein R, Siscovick DS, Rotter JI, Tai ES, Vingerling J, Wong TY: Genome-wide association study of retinopathy in individuals without diabetes. PLoS One 2013;8:e54232

8. Craig DW, Millis MP, DiStefano JK: Genome-wide SNP genotyping study using pooled DNA to identify candidate markers mediating susceptibility to end-stage renal disease attributed to Type 1 diabetes. Diabetic medicine : a journal of the British Diabetic Association 2009;26:1090-1098

9. Greene CN, Keong LM, Cordovado SK, Mueller PW: Sequence variants in the PLEKHH2 region are associated with diabetic nephropathy in the GoKinD study population. Hum Genet 2008;124:255-262

10. Hanson RL, Craig DW, Millis MP, Yeatts KA, Kobes S, Pearson JV, Lee AM, Knowler WC, Nelson RG, Wolford JK: Identification of PVT1 as a candidate gene for end-stage renal disease in type 2 diabetes using a pooling-based genome-wide single nucleotide polymorphism association study. Diabetes 2007;56:975-983

11. Maeda S, Osawa N, Hayashi T, Tsukada S, Kobayashi M, Kikkawa R: Genetic variations associated with diabetic nephropathy and type II diabetes in a Japanese population. Kidney Int Suppl 2007:S43-48

12. Maeda S, Kobayashi MA, Araki S, Babazono T, Freedman BI, Bostrom MA, Cooke JN, Toyoda M, Umezono T, Tarnow L, Hansen T, Gaede P, Jorsal A, Ng DP, Ikeda M, Yanagimoto T, Tsunoda T, Unoki H, Kawai K, Imanishi M, Suzuki D, Shin HD, Park KS, Kashiwagi A, Iwamoto Y, Kaku K, Kawamori R, Parving HH, Bowden DW, Pedersen O, Nakamura Y: A single nucleotide polymorphism within the acetyl-coenzyme A carboxylase beta gene is associated with proteinuria in patients with type 2 diabetes. PLoS Genet 2010;6:e1000842

13. McDonough CW, Palmer ND, Hicks PJ, Roh BH, An SS, Cooke JN, Hester JM, Wing MR, Bostrom MA, Rudock ME, Lewis JP, Talbert ME, Blevins RA, Lu L, Ng MC, Sale MM, Divers J, Langefeld CD, Freedman BI, Bowden DW: A genome-wide association study for diabetic nephropathy genes in African Americans. Kidney Int 2011;79:563-572

14. McKnight AJ, Currie D, Patterson CC, Maxwell AP, Fogarty DG: Targeted genome-wide investigation identifies novel SNPs associated with diabetic nephropathy. Hugo J 2009;3:77-82

15. Pezzolesi MG, Poznik GD, Mychaleckyj JC, Paterson AD, Barati MT, Klein JB, Ng DP, Placha G, Canani LH, Bochenski J, Waggott D, Merchant ML, Krolewski B, Mirea L, Wanic K, Katavetin P, Kure M, Wolkow P, Dunn JS, Smiles A, Walker WH, Boright AP, Bull SB, Doria A, Rogus JJ, Rich SS, Warram

JH, Krolewski AS: Genome-wide association scan for diabetic nephropathy susceptibility genes in type 1 diabetes. Diabetes 2009;58:1403-1410

16. Sandholm N, Salem RM, McKnight AJ, Brennan EP, Forsblom C, Isakova T, McKay GJ, Williams WW, Sadlier DM, Makinen VP, Swan EJ, Palmer C, Boright AP, Ahlqvist E, Deshmukh HA, Keller BJ, Huang H, Ahola AJ, Fagerholm E, Gordin D, Harjutsalo V, He B, Heikkila O, Hietala K, Kyto J, Lahermo P, Lehto M, Lithovius R, Osterholm AM, Parkkonen M, Pitkaniemi J, Rosengard-Barlund M, Saraheimo M, Sarti C, Soderlund J, Soro-Paavonen A, Syreeni A, Thorn LM, Tikkanen H, Tolonen N, Tryggvason K, Tuomilehto J, Waden J, Gill GV, Prior S, Guiducci C, Mirel DB, Taylor A, Hosseini SM, Parving HH, Rossing P, Tarnow L, Ladenvall C, Alhenc-Gelas F, Lefebvre P, Rigalleau V, Roussel R, Tregouet DA, Maestroni A, Maestroni S, Falhammar H, Gu T, Mollsten A, Cimponeriu D, Ioana M, Mota M, Mota E, Serafinceanu C, Stavarachi M, Hanson RL, Nelson RG, Kretzler M, Colhoun HM, Panduru NM, Gu HF, Brismar K, Zerbini G, Hadjadj S, Marre M, Groop L, Lajer M, Bull SB, Waggott D, Paterson AD, Savage DA, Bain SC, Martin F, Hirschhorn JN, Godson C, Florez JC, Groop PH, Maxwell AP: New susceptibility loci associated with kidney disease in type 1 diabetes. PLoS Genet 2012;8:e1002921

17. Tanaka N, Babazono T, Saito S, Sekine A, Tsunoda T, Haneda M, Tanaka Y, Fujioka T, Kaku K, Kawamori R, Kikkawa R, Iwamoto Y, Nakamura Y, Maeda S: Association of solute carrier family 12 (sodium/chloride) member 3 with diabetic nephropathy, identified by genome-wide analyses of single nucleotide polymorphisms. Diabetes 2003;52:2848-2853

18. Tong Z, Yang Z, Patel S, Chen H, Gibbs D, Yang X, Hau VS, Kaminoh Y, Harmon J, Pearson E, Buehler J, Chen Y, Yu B, Tinkham NH, Zabriskie NA, Zeng J, Luo L, Sun JK, Prakash M, Hamam RN, Tonna S, Constantine R, Ronquillo CC, Sadda S, Avery RL, Brand JM, London N, Anduze AL, King GL, Bernstein PS, Watkins S, Jorde LB, Li DY, Aiello LP, Pollak MR, Zhang K: Promoter polymorphism of the erythropoietin gene in severe diabetic eye and kidney complications. Proc Natl Acad Sci U S A 2008;105:6998-7003

19. Lu Y, Ge Y, Hu Q, Shi Y, Xue C, Chen S, Huang Z: Association between angiotensin-converting enzyme gene polymorphism and diabetic retinopathy in the Chinese population. J Renin Angiotensin Aldosterone Syst 2012;

20. Fujisawa T, Ikegami H, Kawaguchi Y, Hamada Y, Ueda H, Shintani M, Fukuda M, Ogihara T: Meta-analysis of association of insertion/deletion polymorphism of angiotensin I-converting enzyme gene with diabetic nephropathy and retinopathy. Diabetologia 1998;41:47-53

21. Abhary S, Hewitt AW, Burdon KP, Craig JE: A systematic meta-analysis of genetic association studies for diabetic retinopathy. Diabetes 2009;58:2137-2147

22. Zhou JB, Yang JK: Angiotensin-converting enzyme gene polymorphism is associated with proliferative diabetic retinopathy: a meta-analysis. Acta Diabetol 2010;47:187-193

23. Niu W, Qi Y, Wu Z, Liu Y, Zhu D, Jin W: A meta-analysis of receptor for advanced glycation end products gene: Four well-evaluated polymorphisms with diabetes mellitus. Mol Cell Endocrinol 2012;

24. Yuan D, Liu Q: Association of the Receptor for Advanced Glycation End Products Gene Polymorphisms with Diabetic Retinopathy in Type 2 Diabetes: A Meta-Analysis. Ophthalmologica 2012;

25. Hu C, Zhang R, Yu W, Wang J, Wang C, Pang C, Ma X, Bao Y, Xiang K, Jia W: CPVL/CHN2 genetic variant is associated with diabetic retinopathy in Chinese type 2 diabetic patients. Diabetes 2011;60:3085-3089

26. Zintzaras E, Chatzoulis DZ, Karabatsas CH, Stefanidis I: The relationship between C677T methylenetetrahydrofolate reductase gene polymorphism and retinopathy in type 2 diabetes: a meta-analysis. J Hum Genet 2005;50:267-275

27. Niu W, Qi Y: An updated meta-analysis of methylenetetrahydrofolate reductase gene 677C/T polymorphism with diabetic nephropathy and diabetic retinopathy. Diabetes Res Clin Pract 2012;95:110-118

28. Zhao S, Li T, Zheng B, Zheng Z: Nitric oxide synthase 3 (NOS3) 4b/a, T-786C and G894T polymorphisms in association with diabetic retinopathy susceptibility: A meta-analysis. Ophthalmic genetics 2012;

29. Ma J, Li Y, Zhou F, Xu X, Guo G, Qu Y: Meta-analysis of association between the Pro12Ala polymorphism of the peroxisome proliferator-activated receptor-gamma2 gene and diabetic retinopathy in Caucasians and Asians. Mol Vis 2012;18:2352-2360

30. Zhang T, Pang C, Li N, Zhou E, Zhao K: Plasminogen activator inhibitor-1 4G/5G polymorphism and retinopathy risk in type 2 diabetes: a meta-analysis. BMC Med 2013;11:1

31. Tian C, Fang S, Du X, Jia C: Association of the C47T polymorphism in SOD2 with diabetes mellitus and diabetic microvascular complications: a meta-analysis. Diabetologia 2011;54:803-811

32. Syreeni A, El-Osta A, Forsblom C, Sandholm N, Parkkonen M, Tarnow L, Parving HH, McKnight AJ, Maxwell AP, Cooper ME, Groop PH: Genetic examination of SETD7 and SUV39H1/H2 methyltransferases and the risk of diabetes complications in patients with type 1 diabetes. Diabetes 2011;60:3073-3080

33. Qiu M, Xiong W, Liao H, Li F: VEGF -634G>C polymorphism and diabetic retinopathy risk: A meta-analysis. Gene 2013;518:310-315

34. Zhao T, Zhao J: Association between the -634C/G polymorphisms of the vascular endothelial growth factor and retinopathy in type 2 diabetes: a meta-analysis. Diabetes Res Clin Pract 2010;90:45-53

35. Gong JY, Sun YH: Association of VEGF gene polymorphisms with diabetic retinopathy: a meta-analysis. PLoS One 2013;8:e84069

36. Lu Y, Ge Y, Shi Y, Yin J, Huang Z: Two polymorphisms (rs699947, rs2010963) in the VEGFA gene and diabetic retinopathy: an updated meta-analysis. BMC Ophthalmol 2013;13:56
